# Optic neuropathy as a presenting feature of vitamin B-12 deficiency: A systematic review of literature and a case report

**DOI:** 10.1016/j.amsu.2020.11.010

**Published:** 2020-11-05

**Authors:** Fateen Ata, Ammara Bint I Bilal, Saad Javed, Hammad Shabir Chaudhry, Rohit Sharma, Rubab Fatima Malik, Hassan Choudry, Anand Bhaskaran Kartha

**Affiliations:** a Department of Internal Medicine, Hamad General Hospital, Hamad Medical Corporation, Doha, Qatar; b Department of Radiology, Hamad General Hospital, Hamad Medical Corporation, Doha, Qatar; c Department of Internal Medicine, Allama Iqbal Medical College, Lahore, Pakistan; d Department of Internal Medicine, Punjab Medical College, Faisalabad, Pakistan; e Weill Cornell Medicine, Qatar

**Keywords:** Vitamin B – 12 deficiency, Cyanocobalamin deficiency, Optic neuritis, Optic nerve, Optic neuropathy, Ophthalmological manifestations, Visual symptoms

## Abstract

**Introduction:**

Vitamin B12 (VitB12) deficiency rarely manifests with visual symptoms. Optic nerve damage in VitB12 deficiency is thought to be via degeneration. However, optic neuritis, though infrequent, has been reported secondary to VitB12 deficiency.

**Material and methods:**

We conducted a systematic review of all the reported cases of VitB12 deficiency with optic nerve involvement in Pubmed, Cochrane, and Google Scholar any date up to September 6, 2020. We have discussed the findings and compiled the available information on ophthalmological manifestations of VitB12 deficiency. We aim to provide a unified knowledge about the evidence related to types of optic neuropathies reported to date secondary to VitB12 deficiency. We also present a case of bilateral optic neuritis secondary to VitB12 deficiency.

**Presentation of case:**

We present a 29-year-old previously healthy male with progressive, painful, bilateral, but asymmetric visual deterioration for forty-five days. A detailed history, examination, and laboratory workup were carried out. He was diagnosed as having optic neuritis secondary to VitB12 deficiency. He showed partial improvement with the replacement of VitB12.

**Conclusion:**

We suggest promptly identifying and replacing VitB12 in patients with optic neuritis with proven VitB12 deficiency to prevent permanent damage to the optic nerve. Patients with VitB12 deficiency should have a baseline fundoscopic exam to rule out subclinical optic nerve damage. Moreover, patients who present with visual disturbances should be screened for VitB12 deficiency, especially the vegan population.

## Highlights

• B 12 deficiency can present with optic neuropathy as an initial manifestation.
• B 12 deficiency-induced optic neuropathy may be reversible if identified timely.
• In patients with visual disturbance, B12 levels should be checked with a fundus exam.
• A subclinical optic neuropathy should be ruled out in B12 deficient patients.

## 1 Introduction

Ophthalmologic involvement is considered a rare manifestation of VitB12 deficiency. It can be a presenting feature or one of the neurological complications of the deficiency. The majority of cases reported in the literature are optic atrophy without optic nerve inflammation [[1], [2], [3], [4], [5], [6], [7], [8], [9], [10], [11], [12], [13], [14], [15], [16], [17]]. However, two cases described optic neuritis in association with VitB12 deficiency [4,12]. Like other neurological manifestations of VitB12 deficiency, ophthalmological involvement is relatively less understood. However, the literature review gives insight into its potential reversibility.

There are many unanswered questions with optic nerve damage secondary to VitB12 deficiency; what is the exact mechanism of optic nerve damage in this condition? Is it reversible or irreversible? Furthermore, how to manage it? Understanding these aspects is imperative, as loss of vision carries a significant morbidity burden, especially secondary to a preventable cause. This review aims to:

a) Gather and discuss in detail the available literature on the ophthalmologic manifestations of VitB12 to provide a pooled knowledge base and access to information for clinical guidance as well as further prospective studies.

b) Discuss essential elements of reported cases, including the presentation and type of optic nerve damage, reversibility of the symptoms, treatments used, and the role of drugs other than vitamin replacement.

## 2 Methods

A literature search was performed for articles using PubMed, Cochrane, and Google Scholar for any date up to September 6, 2020, and all articles in English were analyzed by two authors individually (FA and SJ). The following search term was used “optic atrophy”, “optic neuritis”, “optic neuropathy”, “optic neuropathies”, “ophthalmologic”, “ophthalmic manifestations”, “visual disturbance”, “Visual loss”, “cyanocobalamin deficiency”, “cobalamin deficiency”, “Vitamin B - 12 deficiency”. Search results were initially reviewed by title and abstract. Subsequently, articles were selected for more detailed review and included in the study if deemed relevant. Inclusion criteria were any case report or case series discussing ophthalmic manifestations of vitamin B12 deficiency, and exclusion criteria included reports discussing non-ophthalmic manifestations of B12 deficiency or articles written in a language other than English. Statistical analysis was done for differences in outcome in terms of age, vitamin B12 levels, and gender. Fisher's Exact test, T-tests were applied, and a *P*-value <0.05 cutoff was set as our alpha. SPSS V26 was used for the analysis. A favorable outcome, resolved, was defined as the cases who showed complete or partial improvement, and unresolved were those who lost sight completely or showed no improvement. Our clinical case has been reported according to the SCARE guidelines [18].

## 3 Results

A total of 60 articles (case series and reports) were shortlisted for the initial review. We did not find any prospective clinical trials on the topic. Seven articles were filtered out because they were not in English. Thirty-six articles were removed after the initial analysis as they were not relevant to the topic of discussion and described non-ophthalmological presentations. Seventeen articles were finalized after a detailed review and subsequently added to the manuscript.

Out of the 17 included articles, there are 4 case series and 13 case reports, making 24 cases of VitB12 deficiency with ophthalmological involvement. Among the cases, 8 are females, whereas 16 are males. The median age at presentation is 31 years. Ophthalmological involvement was the initial presentation in twenty-one patients (87.5%), while three patients (12.5%) were known cases of VitB12 deficiency and developed ophthalmological symptoms in due course. The most common presenting complaint was a gradual bilateral decrease in vision. Intramuscular VitB12 replacement followed by oral supplementation was given to twenty-two patients (91.6%), one patient received an only oral replacement, and one patient did not receive VitB12 replacement. Bilateral optic atrophy was the most common diagnosis, seen in 16 patients (66.6%), whereas optic neuritis was diagnosed in two patients. Intravenous steroids were given to three patients (12.5%) in addition to B 12 replacement. A complete resolution of the eye symptoms was seen in six patients (25%), eight patients (33.3%) had partial improvement, seven patients (29%) did not improve, and one patient (4%) progressed to complete blindness. One patient had an improvement of vision in one eye only, and one patient was lost to follow–up. Mean Vit B12 levels were 89.9+- 79.1 and 134.5 +- 44.1 in resolved vs. (versus) unresolved cases. The mean age of the resolved cases was 33 +- 19.7 years vs. 30.1+-17.4 years. None of the differences were statistically significant. 3 out of 7 females showed improvement compared with 13 out of 17 of males, and the difference remained insignificant.

## 4 Our case presentation

A 29-year-old previously healthy Indian gentleman presented with painful, bilateral, but asymmetric visual deterioration for forty-five days. The patient described a gradual progressive reduction in his vision bilaterally to a point where he could not count fingers accurately from 2 m. His vision was deranged more in the right eye compared to the left. He also complained of an intermittent, bifrontal, mild throbbing headache. There was no history of excessive lacrimation, conjunctival redness, trauma to the eyes, or any conjunctival discharge. The patient did not complain of loss of consciousness at any point or intermittent blackouts. The patient had no difficulty in ocular movement. The patient's occupation was soldering, but he used eye protection while at work. A detailed history was taken regarding occupational exposure, drug abuse, metal exposure, or any recent infection but was unrevealing for any cause of his visual impairment. There was no history of sensory or motor dysfunction and no higher cerebral deficits. He did not have any surgical history. The patient did not give any family history of stroke at a young age or multiple sclerosis. There was no family history of ophthalmological disorders. The patient was not taking any medications other than occasional acetaminophen for mild headaches. He did not have any history of psychiatric or mood disorders.

On examination, the patient was afebrile (36.7 °C), normotensive (135/77), with a regular pulse (85 beats per minute) and normal oxygen saturation on room air. Physical exam revealed a well-built male who had markedly reduced visual acuity (unable to count fingers accurately from 2 m). His cranial nerves (other than optic nerve) were intact, and the rest of the physical examination was unremarkable. Extraocular movements were normal and painless. An urgent ophthalmologic exam, including fundoscopy, was arranged, which revealed deteriorated visual acuity and bilateral severe simultaneous optic neuritis. Color vision was also considerably impaired [Table 1]. There was no relative afferent pupillary defect (RAPD) [Table 1].

**Table 1:** Ophthalmologic examination upon admission and 30-day follow-up (IOP: intraocular pressure, RFNL: retinal nerve fiber layer, RAPD: relative afferent pupillary defect).

Measurement	Admission	Follow-up (30 days)
Visual acuity right eye	3/60	6/12
Visual acuity left eye	6/24	6/24
Color vision right eye	1/16	1/16
Color vision left eye	1/16	1/16
Visual field right eye	Normal	Normal
Visual field left eye	Normal	Normal
IOP	17 mmHg	17 mmHg
Conjunctiva/Sclera/cornea	Clear	Clear
RAPD	No RAPD	No RAPD
Fundus right eye	Hyperemic and edematous disc. RFNL thickening Normal vessels No vasculitis Normal periphery	Normal disc RFNL thickening Normal vessels No vasculitis Normal periphery
Fundus left eye	Hyperemic and edematous disc, more than right RFNL thickening, more than right. Normal vessels No vasculitis Normal periphery	Normal disc RFNL thickening Normal vessels No vasculitis Normal periphery

The patient was hospitalized as a case of severe bilateral optic neuritis. Magnetic resonance imaging (MRI) orbit, brain, and spinal cord were performed the same day. MRI scans did not reveal any demyelination or optic nerve enhancement. His complete blood profile and metabolic panel were unremarkable. The antinuclear antibody was negative, ruling out a possibility of vasculitis with optic nerve involvement. Lumbar puncture was done on day two, and cerebrospinal fluid (CSF) was sent for a detailed analysis. The cell count, protein, and glucose were within normal limits, and the CSF was negative for viral, bacterial, or fungal meningitis and tuberculosis. Additionally, there were no oligoclonal bands. The patient was started on intravenous methylprednisolone 1 g for five days. However, the steroids did not improve the vision. At this point, a visual evoked response (VER) was performed, which showed a delayed response, hence reinforcing the presence of optic neuritis.

As the patient was a vegetarian, serum VitB12 level was sent, which was low (80.5 pmol/L, normal range: 145–596 pmol/L). The patient was given intramuscular cyanocobalamin (CNCbl) (1000 mcg daily for seven days) on day 6 of admission and then started on oral CNCbl (1000 mcg once daily). The patient reported mild improvement in vision in the following days.

He was diagnosed with bilateral optic neuritis secondary to VitB12 deficiency because of a detailed negative workup of other causes of optic neuritis, no improvement after pulse steroid therapy, unlike demyelinating diseases like Multiple Sclerosis (MS) or Neuromyelitis Optica (NMO), and evidence of severe VitB12 deficiency with completely normal biochemistry otherwise. He was discharged with a follow-up in the neuro-ophthalmology clinic for a repeat ophthalmologic examination. He was seen 30 days after discharge, and repeated fundoscopic examination revealed resolution of optic neuritis and a slight improvement in vision [Table 1]. The patient was counseled to continue vitamin replacement and was provided with a prescription for a corrective lens. He was followed up once again after 30 days of the first follow-up visit, but this time it was through telecommunication due to COVID-19 restrictions. He stated no further improvement in his vision than his last visit and reported no pain on eye movements. He was compliant with oral Vitamin B 12. The patient could not be followed up further as he lost his job due to the impact of COVID-19 on the local business market and traveled back to his home country.

## 5 Patient perspective

I never had any visual problem before, and this continuous blurring of vision was very worrying for me. I should have come early, but I continued observing it, thinking it to be a usual infection which would subside. I hope that my vision can be restored so that I can work again properly.

## 6 Discussion

Vitamin B – 12, a water-soluble vitamin, is essential for numerous metabolic pathways such as DNA synthesis, RNA synthesis, and DNA methylation. The human body cannot synthesize it, and its levels in the body are dependent entirely on the dietary intake mainly of animal origin such as eggs, milk, and fish, as well as the body's ability to absorb it [19].

### 6.1 Common causes of vitamin B – 12 deficiency

The natural supply of VitB12 is limited to animal products. Therefore, the vegetarian and vegan populations are prone to develop its deficiency [20]. Additionally, pregnant and lactating women are at risk of VitB12 deficiency if animal-related consumption is deficient in their diet [21].

Pathological causes are mainly related to malabsorption due to various reasons, pernicious anemia being the most common amongst them [22]. Other etiologies include gastrectomy, gastritis, Helicobacter Pylori infection, pancreatic insufficiency, small bowel bacterial overgrowth, and fish tapeworm (Diphyllobothrium latum) infestation [23]. Iatrogenic causes encompass partial gastrectomy and medications such as metformin, proton pump inhibitors, nitrous oxide, colchicine, and neomycin [[24], [25], [26]].

### 6.2 Common presentations of vitamin B – 12 deficiency

Vitamin B12 deficiency can manifest as a variety of hematologic abnormalities. The classic finding is deteriorating macrocytic anemia, with pallor and jaundice. Other manifestations can be leukopenia, thrombocytopenia, or even pancytopenia [20,27].

VitB12 deficiency has also been implicated in the pathogenesis of multiple neurological syndromes. The level of morbidity is not related to the extent of the deficiency. Even though coexisting comparable disease severity has been seen, mostly either hematological or neurological complaints prevail. The nervous system can be involved at multiple levels, causing subacute degenerative disorder, peripheral neuropathies, and neuropsychiatric symptoms [28]. Among the cranial nerves, olfactory and optic nerves are most commonly involved [29].

Other systemic manifestations of VitB12 deficiency include generalized pallor, tachycardia, fatigue, palpitations, megaloblastic anemia, unexplained neurological symptoms (dementia, sensory ataxia, personality changes, loss of positional sense), and osteoporosis [5].

Like any disease, patients with VitB12 deficiency can present with atypical manifestations. Some reported findings include dysphagia, hyperpigmentation, psychosis, and acute dementia [[30], [31], [32], [33]].

### 6.3 Ophthalmological manifestations

Reduced visual acuity secondary to VitB12 deficiency is another rarely reported finding. Like other neuropsychiatric manifestations, the current literature on the causative metabolic pathway remains unclear [34,35]. Optic neuropathy is reported to occur in less than 1% of VitB12 deficient patients. It mostly results in progressive, bilateral, painless loss of vision associated with abnormal color vision and central or ceco – central scotomas. The optic nerve may appear normal in the early stages of the disease until optic atrophy develops [5]. Optic atrophy has been reported a few times in the literature, but to the best of our knowledge, optic neuritis is reported only twice before, our case being the third such case [Table 2] [[1], [2], [3], [4], [5], [6], [7], [8], [9], [10], [11], [12], [13], [14], [15], [16], [17]].

**Table 2:** Reported cases of VitB12 deficiency-induced Optic neuropathies (F: female, M: male, OA: Optic Atrophy, ON: Optic Neuritis, NAION: Non-arteritic Anterior Ischemic Optic Neuropathy, NA: not available, OCT: Optical Coherence Tomography, VEP: Visual Evoked Potential, MRI: Magnetic Resonance Imaging, VitB12 normal level: 190 and 950 ng/L).

Author, year	Age, sex	Presentation	Cause of B-12 deficiency	B-12 level ng/L	First symptom	Diagnosing modality + diagnosis	Treatment	Steroids	Outcome
Ellis, P. P et al., 1959 [11]	47, M	Painless bilateral decreased vision, paracentral scotoma in the left eye	NA	NA	No	Fundoscopy Bilateral ON	IM VitB12	Not given	Resolved
de, Olivarius Bf et al., 1961 Case 1 [11]	57, F	Painless bilateral decreased vision, centro-coecal scotomas	NA	105	Yes	Fundoscopy Bilateral OA	IM VitB12	Not given	No Improvement
de, Olivarius Bf et al., 1961 Case 2 [11]	50, F	Painless bilateral decreased vision, Absolute centro-coecal scotomas for red on both eyes	NA	NA	No	Fundoscopy Bilateral OA	IM VitB12	Not given	NA
Björkenheim, B, 1966 Case 1 [9]	41, M	Painless bilateral blurring of vision	Tapeworm infestation	16	Yes	Fundoscopy Bilateral OA	Removal of worm	Not given	Resolved
Björkenheim, B, 1966 Case 2 [9]	19, M	Painless bilateral blurring of vision and bilateral centro-cecal scotomas to white	Tapeworm infestation	21	Yes	Fundoscopy Bilateral OA	Removal of worm	Not given	Resolved
Björkenheim, B, 1966 Case 3 [9]	39, F	Painless bilateral blurring of vision and bilateral centro-cecal scotomas to white	Tapeworm infestation	25	Yes	Fundoscopy Bilateral OA	Removal of worm	Not given	Resolved
Björkenheim, B, 1966 Case 4 [9]	17, M	Painless bilateral blurring of vision and bilateral centro-cecal scotomas to red	Tapeworm infestation	72	Yes	Fundoscopy Bilateral OA	Removal of worm	Not given	No Improvement
Foulds, W. S et al., 1969 Case 1 [3]	71, M	Painless bilateral decreased vision, Bilateral centro-cecal scotoma	Pernicious anemia	46	Yes	NA	IM followed by Oral VitB12	Not given	Improved
Foulds, W. S et al., 1969 Case 2 [3]	56, M	Painless bilateral decreased vision, bilateral centro-cecal scotoma	Pernicious anemia + dietary	25	Yes	Fundoscopy	Oral VitB12	Not given	Resolved
Gleeson, M. H et al., 1974 [13]	19, F	Painless bilateral decreased vision and large central scotoma, initially in left, then bilateral	Dietary	80	Yes	Fundoscopy, Advanced bilateral OA	IM followed by Oral VitB12	Given	Right vision resolved, Left No improvement
Stambolian, D et al., 1977 [17]	17, M	Painless bilateral decreased vision, centro-cecal scotomas to red	Bowel resection	190	Yes	Fundoscopy	IM VitB12	Given	Improved
de Letona, J. M, 1998 [10]	64, M	A painless bilateral progressive loss of visual acuity preceded by dyschromatopsia	Gastro ileal anastomosis	NA	No	Fundoscopy	Surgical correction of gut	Not given	Improved
Koh, A. H et al., 1998 [14]	37, M	Retinopathy	NA	106	Yes	Fundoscopy, Retinopathy	IM VitB12	Not given	Improved
Moschos, M et al., 1998 [16]	55, M	Painless bilateral decreased vision	Gastroplasty	180	Yes	VEP, Bilateral OA	IM VitB12	Not given	No Improvement
Milea, D et al., 2000 [15]	33, M	Painless bilateral impaired vision , central scotomata, dyschromatopsia	Dietary	114	Yes	Fundoscopy, Bilateral OA	IM followed by Oral VitB12	Not given	No Improvement
Larner, A. J, 2004 [5]	29, F	Painless bilateral impaired vision Centro-cecal scotoma.	Pernicious anemia	115	Yes	Fundoscopy, Bilateral OA	IM followed by Oral VitB12	Not given	No Improvement
Pineles, S. L et al., 2010 Case 1 [7]	6, M	Painless bilateral decreased vision	Dietary	150	Yes	MRI, Fundoscopy, Bilateral OA	IM followed by Oral VitB12	Not given	No Improvement
Pineles, S. L et al., 2010 Case 2 [7]	13, M	Painless bilateral decreased vision	Dietary	297	Yes	MRI, Fundoscopy, Bilateral OA	IM followed by Oral VitB12	Not given	Improved
Pineles, S. L et al., 2010 Case 3 [7]	7, M	Painless bilateral decreased vision	Dietary	155	Yes	MRI, Fundoscopy Bilateral OA	IM followed by Oral VitB12	Not given	Improved
Chu, C et al., 2011 [1]	19, M	Asymptomatic	NA	52	Yes	Fundoscopy, VEP, Bilateral OA	IM followed by Oral VitB12	Not given	Improved
Zehetner, C et al., 2011 [8]	40, M	Diminished left visual acuity and a drop-shaped central scotoma	Dietary	40	Yes	Fundoscopy, OCT, Bilateral white centered retinal hemorrhages, Retinal thickening	IM followed by Oral VitB12	Not given	Resolved
Jalil, A et al., 2012 [4]	11, F	Bilateral painless progressive visual loss	Dietary	NA	Yes	MRI, VEP, Bilateral ON progressed to OA	IM followed by Oral VitB12	Given	Progressed to blindness
Dobson, R et al., 2016 [2]	22, F	Painless bilateral decreased vision	Dietary	166	Yes	MRI, VEP, Fundoscopy, Bilateral demyelinating OA	IM followed by Oral VitB12	Not given	Improved
Pantalon, A. D et al., 2016 [6]	22, F	A sudden bilateral painless drop of vision	Dietary	166	Yes	MRI, VEP, Fundoscopy, Bilateral NAION	Nothing	Not given	No Improvement
Our case, 2020	29, M	Painful bilateral loss of vision, pain more in the right eye	Dietary	80.5	Yes	Fundoscopy. VEP, Bilateral ON	IM followed by Oral VitB12	Given	Improved

Although no prospective studies focus on the timing of the ophthalmological involvement and its correlation with the serum level of VitB12, post – mortem fundoscopic examination of patients with pernicious anemia have shown damaged optic nerves. Additionally, Gokce Cokal, B et al. have reported reduced Visual Evoked Potential (VEP) amplitudes in asymptomatic VitB12 deficient patients. This suggests subclinical optic nerve damage preceding symptomatic eye involvement in VitB12 deficiency [36]. Hence, it would be prudent to evaluate cost-effectiveness in screening VitB12 deficiency in asymptomatic patients [37].

The most common presenting ophthalmological manifestation in VitB12 deficiency is optic atrophy, followed by optic neuritis in the literature. Two patients in our literature review also had retinopathy. Retinal hemorrhage was documented on fundus examination in one of them. Another rare ophthalmological manifestation is Non-arteritic Anterior Ischemic Optic Neuropathy (NAION), reported in one patient who presented with a sudden decrease in vision, contrary to most cases where the reduction of vision was gradual, ranging from weeks to months.

The literature suggests posterior segment involvement and sparing of the anterior segment of the eye. In the posterior segment, the most commonly involved part is the optic nerve.

### 6.4 Optic atrophy

Optic atrophy was found in 16 patients [Table 2]. Some of the cases did not mention the type of optic involvement; therefore, they might have had a similar diagnosis. Almost all the patients had a progressively decreasing vision as the presenting complaint. The symptom duration in the cases ranged from weeks to months, indicating a chronic nature of the underlying disease, as expected in nutritional deficiencies. Visual examination revealed reduced visual acuity in all the cases. Most of the patients had centro-cecal scotomas, mainly to white. Bilateral involvement upon presentation was seen in all except one, in whom the left eye was involved first, with eventual involvement of the right eye. The etiology of VitB12 deficiency in most cases was secondary to a restricted diet. One patient had pernicious anemia, one had a gastrectomy, and four had tapeworm infestation. In eight patients, there was either a complete recovery or improvement in the symptoms with CNCbl replacement, while the remaining eight had no improvement in their visual symptoms.

### 6.5 Optic neuritis

Optic neuritis is reported previously in two patients. The first case was a 47 years old male who presented with a painless, bilateral decreased vision with paracentral scotoma in the left eye. The diagnosis was made based on the findings of the fundoscopic exam. The cause of VitB12 deficiency, as well as the level of the vitamin, was not reported. He was treated with Intramuscular (IM) CNCbl and eventually had a complete resolution of his symptoms.

The second patient was 11 years old female who presented with bilateral painless and progressive visual deterioration. Her diagnosis of optic neuritis was based on MRI and VEP findings. Although the VitB12 deficiency was not quantified, the authors mention a very low level upon diagnosis [4]. The patient received Intramuscular and Oral CNCbl replacement and intravenous steroids. However, she progressed to optic atrophy and eventually ended up with complete blindness. The patient's visual complaints started two weeks before the presentation, indicating either the patient may have had a subclinical involvement before that, or the duration of symptoms may not have a role in determining reversibility.

## 7 The pathophysiology behind ophthalmologic involvement in B – 12 deficiency

The exact pathophysiologic mechanism behind optic neuropathy secondary to VitB12 deficiency is unknown. In order to better understand the neuropathology, VitB12 deficient Cd3202/2 mice were studied, demonstrating increased levels of Tumor Necrosis Factor-alpha (TNF-a), a marker for inflammation, in the spinal cord. Activated macrophages and microglia secrete it without a clear cut understanding of the metabolic pathway leading to its release [38].

In another animal study, Chan et al. investigated VitB12 as a superoxide scavenger in female Long-Evans rats. Cobalamin, both in vitro and in vivo, decreased the oxidative stress in the retinal ganglion cells by scavenging superoxides. Additionally, when cells were treated with menadione (to include superoxide stress) both in the presence and absence of CNCbl, the cells with CNCbl had a prolonged survival, compared to the controls [39].

One of the proposed mechanisms of starting this chain of inflammation is non-re-methylation or *trans*-sulfuration of methionine, leading to increased homocysteine levels, a highly reactive amino acid. High levels of homocysteine presumably are toxic to vascular endothelium and injure the vessels by generating free radicals. This is the current understanding of Anterior ischemic optic neuropathy secondary to cobalamin deficiency. We believe a similar pathway may be implicated in the pathogenesis of optic neuritis [40].

### 7.1 Diagnosis

Optic neuritis typically presents with acute, unilateral, painful vision loss, whereas optic atrophy presents a more chronic and painless vision loss. Diagnosis of optic neuropathy secondary to VitB12 deficiency requires detailed patient history, thorough ophthalmologic examination, serum VitB12, folate, homocysteine & methyl-malonyl CoA levels, and Schilling test (historical interest) in addition to optic neuritis-specific investigations. Essential aspects in the patient's history include dietary habits and associated conditions that cause VitB12 deficiency such as Celiac disease, IBD, Bariatric, Gastric, or Intestinal surgery [29].

MRI of the brain and orbits with gadolinium enhancement helps rule out compressive, ischemic, inflammatory, demyelinating, or infiltrative etiologies [41]. Despite several innovations in MRI technology, the affected optic nerves show abnormal enhancement on MRI in 94.4% of cases of optic neuritis, while 5.6% of the cases may not show any enhancement [42].

In atypical presentations such as bilateral, asymmetric visual loss or normal MRI findings, cerebrospinal fluid analysis, fluorescein angiography, VEP, optical coherence tomography, automated visual field assessment, and color vision testing can aid in establishing the diagnosis [43,44].

### 7.2 Treatment

The treatment of optic neuropathy secondary to VitB12 deficiency has many unanswered aspects. Which route and dose of vitamin replacement should be used? Should it depend on the level of serum VitB12 or the extent of the optic nerve damage? Whether there is a role of anti-inflammatory medications such as systemic steroids, especially in the subset of patients with optic neuritis. These are all significant components to be addressed given that the cause is preventable, treatment is readily available in all parts of the world regardless of the status of healthcare, and finally, because the consequences (such as blindness) carry a high morbidity burden.

Prompt vitamin replacement is the mainstay of the treatment. One of the reports mentions a visibly better response with hydroxocobalamin (l000 μg twice per week) compared to CNCbl replacement concerning visual symptoms [3]. Early and aggressive replacement of VitB12 helps in visual recovery, and clinical response is best seen in the first three months of replacement [34,41].

The role of steroids is not established in optic neuritis and neuropathy secondary to VitB12 deficiency; however, it has been used previously, mainly in optic neuritis [4]. Like the previous authors' experience, steroids did not seem to impact the resolution of our patient's visual symptoms. Nevertheless, one of the rationales to administer steroids is when the diagnosis is not confirmed, and there is still a suspicion of steroid-responsive diseases such as MS or NMO. Optic involvement in NMO can precede other manifestations, and sometimes it can be the only presentation, which makes it challenging to avoid steroids in such cases, even when VitB12 deficiency is established [45].

In most cases reported before, the visual symptoms either improved or resolved entirely, whereas a few cases did not improve or even deteriorated [[1], [2], [3], [4], [5], [6], [7], [8], [9], [10], [11], [12], [13], [14], [15], [16], [17]]. The factors associated with the reversibility of the optic nerve damage can be related to patient characteristics, cause of VitB12 deficiency, the severity of symptoms, duration of symptoms, levels of VitB12, and treatment modalities used, among other relatively less obvious determinants.

We believe that this review offers insight into a rare but potentially devastating medical condition. It should be of utmost priority to rectify any nutritional deficiency that may lead to otherwise preventable morbidity. We have highlighted one such condition, i.e., optic nerve damage secondary to VitB12 deficiency. The major limitation of this article is that it includes only small observational studies (case series and reports). More extensive observational and prospective studies will be valuable in gaining insight regarding the unanswered aspects of VitB12 deficiency-induced optic nerve damage.

## 8 Conclusion

VitB12 deficiency should be ruled out in patients who present with visual disturbances, especially in the vegetarian population, as the prevalence of VitB12 deficiency is not uncommon among them. Timely identification of the deficiency and replacement of cyanocobalamin is imperative in preventing permanent optic nerve damage. Optic neuropathy can be the presenting feature of VitB12 deficiency and may be reversible via a prompt identification and replacement of VitB12.

## 9 Recommendations

After our detailed literature review and clinical observations, we recommend the following:

1. Any patient presenting to the Ophthalmology or Medicine clinic with visual disturbance, a detailed fundoscopic examination should be done, and VitB12 levels should be checked, especially if the patient gives a history of a vegetarian diet.
2. If vitamin VitB12 levels are low, or if a patient is vegetarian and not taking multivitamins/VitB12, it should be urgently replaced, and a particular focus should be placed on reversing the ophthalmological damage whenever present.
3. Patients with incidental findings of VitB12 deficiency or those diagnosed with VitB12 deficiency due to non-visual symptoms should have a detailed fundoscopic examination to rule out subclinical optic nerve damage.

## Declaration

This manuscript is original work and has not been submitted or is not under consideration for publication elsewhere. All the authors have reviewed the manuscript and approved it before submission. None of the authors have any conflict of interest from publishing this work.

## Appendix A Supplementary data

Application Application
